# Lower esophageal microbiota species are affected by the eradication of *Helicobacter pylori* infection using antibiotics

**DOI:** 10.3892/etm.2015.2169

**Published:** 2015-01-07

**Authors:** ZHIYING TIAN, ZHIBANG YANG, JIYE GAO, LILI ZHU, RENJU JIANG, YING JIANG

**Affiliations:** 1 Department of Pathogen Biology, School of Basic Medical Sciences, Chongqing Medical University, Chongqing 400016, P.R. China; 2 Laboratory of Pathogen Biology and Immunology, Teaching and Experiment Center of Basic Medicine, Chongqing Medical University, Chongqing 400016, P.R. China

**Keywords:** lower esophagus, microbiota, *Helicobacter pylori*, infection, antibiotics, polymerase chain reaction-denaturing gradient gel electrophoresis

## Abstract

The aim of this study was to investigate the effect of *Helicobacter pylori* (*H. pylori*) infection on the lower esophageal microbiota and the eradication of *H. pylori* through the use of antibiotics. Forty-five BALB/C mice were randomly divided into negative control, infection and treatment groups. The mice were sacrificed and DNA was extracted from the lower esophageal microbiota. Polymerase chain reaction-denaturing gradient gel electrophoresis (PCR-DGGE) was performed to determine the composition of the microbiota. Quantity One^®^ 1-D Analysis Software was used for the analysis of the DGGE profiles. The different bands from the groups were amplified with 16S rDNA V6 region primers. DNA sequencing and Basic Local Alignment Search Tool analysis were performed for the identification of the bands. *H. pylori* colonization led to severe ulcers in the stomachs of the mice, and these ulcers were alleviated by antibiotic treatment. The infection group had an increased number of bacterial species in the stomach compared with the control and treatment groups. DGGE fingerprinting of the lower esophagus showed that there were significant differences in the number of bands (P<0.05), diversity index and abundance among the groups (P<0.05); however, no significant differences in homogeneity were observed (P>0.05). Although the composition of flora species in the lower espohagus varied, the dominant species, and their relative contents, were similar in the control, infection and treatment groups. The present study provided a microecological basis for the understanding of the pathogenesis of lower esophageal diseases, following the eradication of *H. pylori* infection with antibiotics.

## Introduction

*Helicobacter pylori* (*H. pylori*) are one of the major risk factors for stomach disease. Routine clinical treatments for clearing *H. pylori* infection are usually triple or quadruple antibiotic therapies, but they are often accompanied with increased incidence of esophageal diseases (1).

Studies have shown that the incidence of reflux esophagitis in patients with gastroduodenal ulcer and *H. pylori* infection who were treated with antibiotic therapy (26%) was higher than that in patients who did not receive antibiotic therapy (13%). It was reported that reflux esophagitis often occurs following *H. pylori* eradication therapy (2–6), and that malignant esophageal adenoma is a complication of reflux esophagitis. A retrospective examination carried out in patients with Barrett’s esophagus showed that, following *H. pylori* eradication therapy, the columnar epithelium area expanded into the area of the gastroesophageal junction (7). In 2011, a study in Japan reported the appearance of lower esophageal malignant adenomas in a patient with stomach ulcers following *H. pylori* eradication therapy (7). These studies suggested that the use of antibiotic therapy for the eradication of *H. pylori* may increase esophageal disease, and *H. pylori* may become a potential risk factor for esophageal cancer.

It has been found that simian immunodeficiency virus infection can cause significant changes in the chimpanzee intestinal microbiota (8). Furthermore, in a previous study, the 16S rRNA sequencing of intestinal microbiota was carried out for individuals with human immunodeficiency virus (HIV)-negative infection, new HIV-1 infection and old HIV-1 infection. The results showed that HIV infection was associated with unique changes in the intestinal microbiota. Antiviral therapy did not allow the microbial communities to return to the HIV-negative status. HIV-infected intestines had the characteristics of chronic intestinal enteritis, but the similarity of the HIV-associated microbiota to the microbiota of other inflammatory states was limited, which increased the diversity (9).

As an important part of the esophageal microenvironment, the microbiota maintains the stability and balance of the microenvironment through the regulation of various systems (10). The lower esophagus is closely connected to the stomach. Changes in the composition of the lower esophageal microbiota can be caused by the colonization of *H. pylori* in the stomach; thus, the association between these changes and diseases in the lower esophagus warrants investigation. In the present study, mouse models were used to analyze the changes in the composition of the lower esophageal microbiota following *H. pylori* infection and the eradication of the *H. pylori* by antibiotics. In addition, the mechanisms of diseases in the lower esophagus were further investigated.

## Materials and methods

### Reagents and equipment

*H. pylori* standard strain (Hp11637) was obtained from the Department of Clinical Microbiology, Third Military Medical Universiry (Chongqing, China), and was stored in the Laboratory of Pathogen Biology and Immunology (Chongqing, China). Omeprazole was purchased from Shanxi Jinhua Huixing Pharmaceutical Co., Ltd. (Yuncheng, China). The antibiotics ampicillin and clarithromycin were purchased from Zhangjiajie Yuan Pharmaceutical Co., Ltd. (Zhangjiajie, China) and Xingtai Mingshen Pharmaceutical Factory (Xingtai, China), respectively. The bacterial DNA extraction kit was provided by Tiangen Biotech (Beijing) Co., Ltd. (Beijing, China). Polymerase chain reaction (PCR) reagents were purchased from Takara Biotech Inc. (Dalian, China). PCR-denaturing gradient gel electrophoresis (DGGE) instruments and all associated equipment were purchased from Bio-Rad (Hercules, CA, USA). The primer sequences specific for the *H. pylori* 16S rDNA were as follows: Reverse, 5′-TTTGTTAGAGAAGATAATGACGGTATCTAA-3′; forward, 5′-CATAGGATTTCACACCTGACTGACTATC-3′. The primer sequences for the prokaryotic rDNA V6 region containing the GC clamp were as follows: Reverse, 5′-CGGTGTGTACAAGACCC-3′; forward, 5′-CGCCCGGGGCGCGCCCCGGGCGGGGCGGGGGCACGGGGGCACGGGGGGAACGCGAAGAACCTTAC-3′. The primer sequences for the prokaryotic rDNA V6 region without the GC clamp were as follows: Reverse, 5′-CGGTGTGTACAAGACCC-3′; forward, 5′-AACGCGAAGAACCTTAC-3′. All primers were synthesized by Shanghai Yingjun Biotechnology Company (Shanghai, China).

### Animals

Specific pathogen-free female BALB/c mice (aged 6–8 weeks and weighing 18–20 g), provided by the Experimental Animal Center of Chongqing Medical University (Chongqing, China), were randomly divided into three groups of 15 mice. Group A was the negative control group, which was not infected with *H. pylori*, while the mice in group B were infected with *H. pylori* (infection group). The mice in group C were treated with antibiotics subsequent to being infected with *H. pylori* (treatment group). Following fasting for 12 h, the mice in groups B and C were administered 0.5 ml 2×10^9^ CFU/ml fresh *H. pylori* solution via gavage, which was repeated every three days for five times in total. After an interval of four weeks, group C was fasted for 12 h each day and then orally administered 0.75 ml solution containing 0.25 ml 0.2 mg/ml omeprazole, 0.25 ml 20 mg/ml ampicillin and 0.25 ml 50 mg/ml clarithromycin. The mice were subsequently fasted for a further 3–4 h. These processes were carried out once a day for seven consecutive days. Similarly, the mice in group B were treated in the same manner using sterile saline instead of antibiotics. In group A, sterile saline solution was used to replace the *H. pylori* bacteria and antibiotics, but all other processes were identical. The mice in the three groups were sacrificed at the same time, once the mice in group C had been treated with antibiotics for two weeks. All animal experiments were conducted according to the ethical guidelines of Chongqing Medical University.

### Colonization of H. pylori

Following the sacrifice of the mice by decapitation, the entire stomach of each mouse was washed and divided into two parts. One half was tested with a rapid urease test strip, in which a change from yellow to blue was judged to be a positive result. The rapid urease test strip was produced according to the methods of a previous study (11). The other half was manually ground in 500 μl sterile saline, and more sterile saline was added to produce a total volume of 1 ml, from which 100 μl was taken for streaking inoculation on *H. pylori*-selective agar plates. The inoculated agar plates were placed in an anaerobic jar in which a microaerophilic environment was formed using airbags at 37°C for 74 h.

The remaining homogenate was used for DNA extraction with a bacterial DNA extraction kit. DNA extractions were performed according to the manufacturer’s instructions. The primer sequences specific for the *H. pylori* 16S rDNA were then used for PCR amplification of the extracted DNA. The PCR conditions were set as follows: Initial denaturation at 95°C for 10 min; 30 cycles of 95°C for 30 sec (denaturation), 60°C for 30 sec (annealing) and 72°C for 30 sec (extension), and a final extension step at 72°C for 10 min. The extracted *H. pylori* DNA was used as positive control. The amplification products were analyzed using agarose gel electrophoresis. *H. pylori* colonization was considered to have occurred if two of the following three tests had positive results: Isolation culture bacteria, rapid urease test and PCR analysis. In the treatment group, *H. pylori* eradication was considered to have occurred if the results for *H. pylori* culture, rapid urease test and PCR analysis were all negative.

### Pathohistological observation

The mice were decapitated and the stomachs were retrieved under sterile conditions for visual inspection of the control and infected stomachs. The residues inside the stomachs were washed off by sterile saline. The stomachs were longitudinally cut open for ulcer observation. According to the clinical criteria for judging the degree of gastric mucosal lesions (12), the pathological stomach changes in the infection and treatment groups were classified as follows: No pathological changes, inflammation, mild ulcers and large ulcers (ulcer diameter >2 cm).

### Hematoxylin-eosin (HE) staining

Tissues of the lower esophagus were fixed with 10% formaldehyde, dehydrated by a graded ethanol series, embedded by paraffin and sliced into serial vertical sections. Finally, the slices were stained by HE for observation under a microscope.

### PCR-DGGE

Following the sacrifice of the mice, one-third of the lower esophagus was removed under sterile conditions, and further cut longitudinally into two halves. One half was fixed using 10% formaldehyde solution and was observed under the microscope subsequent to HE staining. The other half was homogenized for bacterial DNA extraction using a DNA extraction kit. The extracted DNA was used as a template, and primers for the prokaryotic 16S rDNA V6 region containing a GC clamp were used to amplify the bacterial genomic DNA (13). The amplification products were analyzed by DGGE using the Bio-Rad gel irrigation system (Quantity One 1-D Analysis Software version 4.6.2; Bio-Rad Laboratories, Inc., Hercules, CA, USA), in which the denaturing gradient of vertical electrophoresis was 0–80%, the temperature was maintained at 60°C, the voltage was 85 V and the total time of electrophoresis was 16 h. The gel was rinsed with deionized water and stained with silver nitrate subsequent to electrophoresis, and the GD7500 gel imaging analysis system (Bio-Rad) was then used for imaging.

### DGGE profiling

DGGE patterns for each group were analyzed. Three samples were randomly selected from group A, and nine samples were randomly selected from groups B and C, respectively. PCR-DGGE was performed for a total of 21 samples on the same piece of gel using Quantity One^®^ 1-D Analysis Software (Version 4.6.2; Bio-Rad). Following standard processing, the DGGE staining patterns were analyzed. The Shannon-Weiner diversity index (14) was calculated using the following formula: H = −∑ (Ni/N) × ln (Ni/N), in which Ni was the brightness of the individual bands and N was the brightness of all the bands. The homogeneity (Pielou) index (14) was calculated using the formula E = H′/lnS, in which S was the number of the bands, and the abundance (Margalef) index (14) was calculated using the formula R = (S−1)/lnN. According to the criteria provided by Jiang *et al* (15), the average value of OD_302_50 was taken as the cut-off value to analyze the average number of bands for each experimental group. The Student’s t-test was used to examine statistically significant differences between group bands.

Quantity One software was used to analyze the similarity of profiles between different groups. Data were processed using the unweighted pair group method with arithmetic mean, and tree diagrams for similarity between groups were plotted. The similarity of the microbiota composition in the lower esophagus was then analyzed on the basis of the tree diagrams.

### Sequencing analysis

The recycled amplification products were sent for sequencing and the DNA sequences were determined using Basic Local Alignment Search Tool (BLAST) (http://blast.ncbi.nlm.nih.gov/Blast.cgi) identification (16).

### Statistical analysis

All statistical analyses were performed using SPSS 17.0 for Windows (SPSS Inc., Chicago, IL, USA). Results are expressed as the mean ± standard deviation. P<0.05 was considered to indicate a statistically significant difference.

## Results

### H. pylori colonization leads to severe ulcers in the mouse stomach, which are alleviated by antibiotic treatment

**Figure 1:**
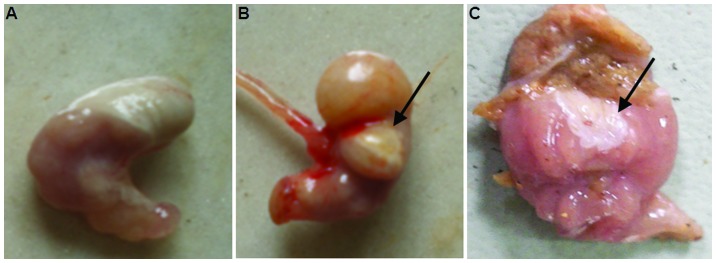
Visual inspection of (A) normal mouse stomach, (B) infected mouse stomach and (C) mouse stomach with large ulcers. The mice were decapitated and the stomachs were retrieved under sterile conditions for the visual inspection of the control and infected stomachs. The residues inside the stomachs were washed off by sterile saline. The stomachs were longitudinally cut open for ulcer observation. The arrows indicate ulcers.

To test the effect of antibiotic treatment on *H. pylori* infection, *H. pylori* colonization in the mouse stomach was measured and the stomachs were visually inspected. The results showed no *H. pylori* colonization in the negative control group, while the percentage of colonization in the infection group and treatment group was 100%, respectively; the *H. pylori* eradication rate was 93% in the treatment group (Table I). Visual inspection showed that 93% of the mice in the infection group had gastric lesions, the majority of which were classified as large ulcers (Fig. 1 and Table II). Following treatment, the percentage of mice with gastric lesions did not change, but the ulcers were mitigated (Table II). These data indicated that *H. pylori* infection had a serious effect on the stomachs of mice, and that treatment with antibiotics reduced the degree of gastric ulceration.

### Antibiotic treatment reduces gastric lesions induced by H. pylori infection according to histological examination results

**Figure 2:**
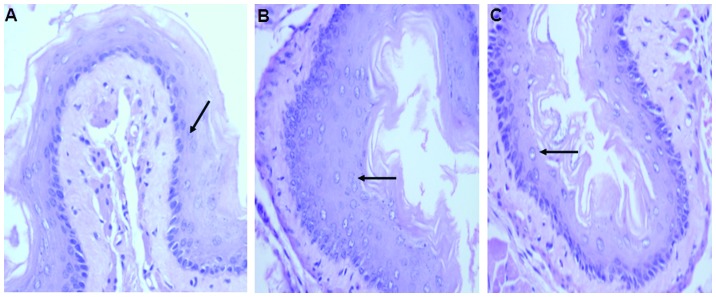
Tissue sections of a mouse stomach from (A) the negative control group, (B) the infection group and (C) the treatment group. Following the sacrifice of the mice, one-third of the lower esophagus was removed under sterile conditions and further cut longitudinally into two halves. One half was fixed using 10% formaldehyde solution and observed under the microscope following hematoxylin and eosin staining (magnification, ×40). The arrows indicate inflammatory cells.

To visualize how antibiotic treatment affected the *H. pylori*-infected mouse stomach, HE staining and microscopy were performed. The results showed mucosal integrity, no damage and few inflammatory cells in the negative control group (Fig. 2A). By contrast, mucosal injury, increased number of glands and significantly increased inflammatory cell infiltration were observed in the infection group (Fig. 2B). In the treatment group, severe mucosal injury and glandular atrophy were visualized, but the number of inflammatory cells was significantly decreased (Fig. 2C). These results suggested that antibiotic treatment attenuated the gastric lesions induced by *H. pylori* infection.

### PCR amplifies the bacterial 16S rDNA V6 region

**Figure 3:**
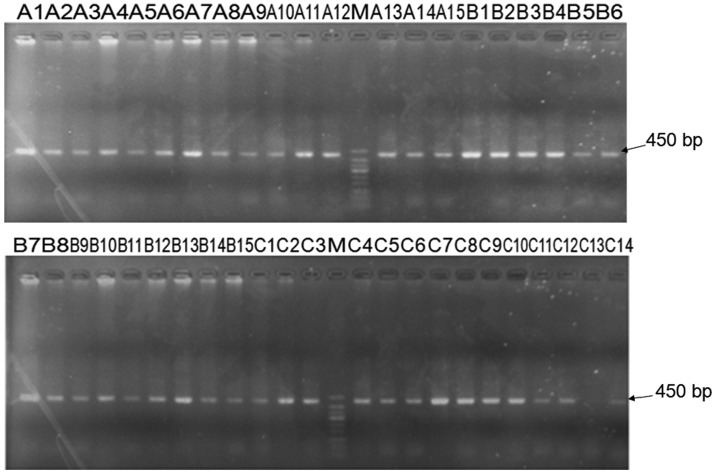
Agarose gel electrophoresis analysis of 16S rDNA amplification products by polymerase chain reaction. Following the sacrifice of the mice, one-third of the lower esophagus was removed under sterile conditions and further cut longitudinally into two halves. One half was homogenized for bacteria DNA extraction using a DNA extraction kit. The extracted DNA was used as a template, and primers specific to the prokaryotic 16S rDNA V6 region containing a GC clamp were used amplify bacterial genomic DNA. The amplification products were analyzed by agarose gel electrophoresis analysis. A, negative control group; B, infection group; C, treatment group.

To analyze the PCR amplification products, agarose gel electrophoresis was performed. The data showed that the bacterial DNA extracted from the lower esophagus was specifically amplified by PCR, with the amplified fragment length of ~450 bp. This observation was consistent with the expected amplified fragment length (Fig. 3), which suggested that the 16S rDNA V6 region was correctly amplified.

### Increased numbers of bacterial species are observed in the stomachs of the infection group compared with the stomachs of the control and treatment group mice

**Figure 4:**
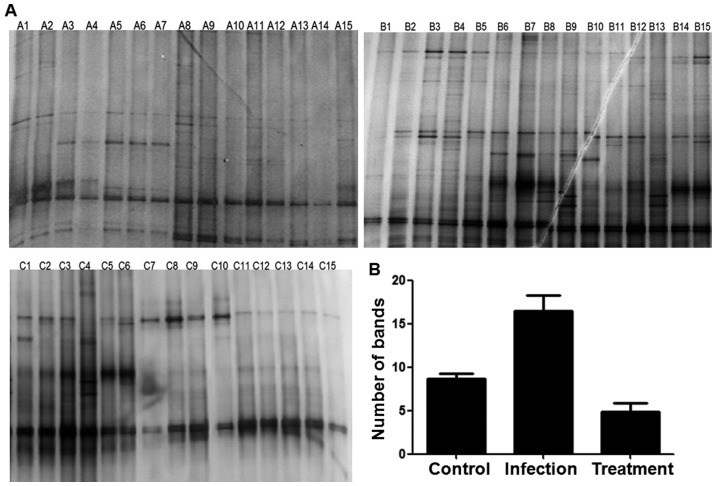
DGGE profiles in the lower esophagi of mice. (A) 16S rDNA separated by DGGE from lower esophageal bacteria. Each lane represents the flora DGGE profile for each mouse, and each band represents the 16S rDNA V6 region of one species of bacterium. The number of bands in the same lane corresponds to the species number of flora in the lower esophagus of the mouse. (B) Quantification of the average number of bands in the three groups. The histogram represents the number of bands for groups A, B and C. Data are expressed as the mean ± standard deviation. The Student’s t-test showed significant differences in the number of bands between any two groups (P<0.05). DGGE, denaturing gradient gel electrophoresis; A, negative control group; B, infection group; C, treatment group.

To determine the number of bacterial species present in the microbiota in the three groups of mice, DGGE profiling was performed. The DGGE profiles in the lower esophagus showed that the numbers of bands in groups A, B, and C were 8.7±0.6, 16.4±1.8, and 4.9±0.9, respectively (Fig. 4). These data indicated that more species were present in the infection group than in the control and treatment groups.

### Bacterial species in the lower esophagus vary among the three groups of mice, but the dominant species and the relative content are similar

**Figure 5:**
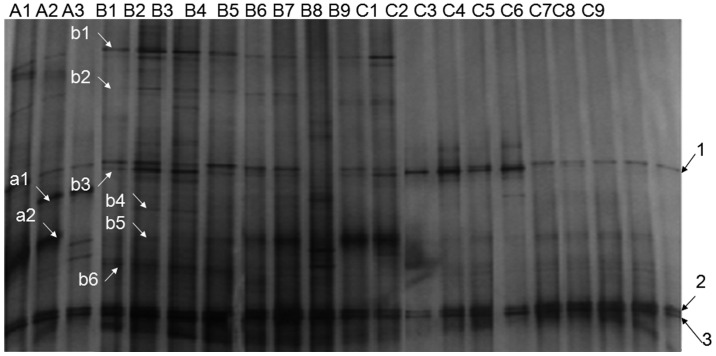
DGGE profiles of the lower esophageal bacterial 16S rDNA in selected mice. The amplification products of the bacterial 16S rDNA V6 were analyzed by DGGE using the Bio-Rad (Hercules, CA, USA) gel irrigation system, in which the denaturing gradient of vertical electrophoresis was 0–80%, the temperature was maintained at 60°C, the voltage was 85 V and the total time of electrophoresis was 16 h. The gel was rinsed with deionized water and stained with silver nitrate subsequent to electrophoresis, and the GD7500 gel imaging analysis system (Bio-Rad) was then used for imaging. Each lane represents a mouse, each band represents a type of bacteria, and the number of bands in the same lane corresponds to the number of bacterial species. DGGE, denaturing gradient gel electrophoresis; A, negative control group; B, infection group; C, treatment group.

To analyze the diversity of the bacterial species in the lower esophageal microbiota of mice, diversity index calculation methods were used to investigate the number of bands and gray scales for each of the groups in the DGGE fingerprints. The analysis showed that there were significant differences in the diversity and abundance indices of the DGGE profiles among the three groups (P<0.05); however, the homogeneity index showed no significant differences among the groups (P>0.05) (Table III). The abundance index reflected the number of bacterial species present, while the diversity index indicated the heterogeneity among the groups and the homogeneity index reflected the dominant species present in the microbiota and the relative content (Fig. 5). These results suggested that the bacterial species in the lower esophageal microbiota varied among the three groups of mice, but the dominant species and the relative content were similar.

### H. pylori infection and antibiotic treatment can change the composition of the stable microbial community in the lower esophagus

**Figure 6:**
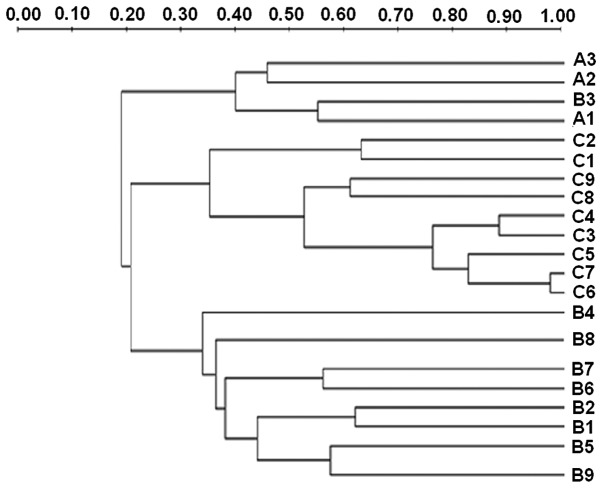
Analysis of PCR-DGGE patterns of bacteria in each group. Three samples were randomly selected from group A, and nine samples were randomly selected from groups B and C, respectively. PCR-DGGE was performed for a total of 21 samples on the same piece of gel. Quantity One^®^ software (Bio-Rad, Hercules, CA, USA) was used to analyze the similarity of profiles between different groups. Data were processed using the unweighted pair group method with arithmetic mean, and tree diagrams for similarity between groups were plotted. The similarity of the microbiota composition in the lower esophagus was then analyzed on the basis of the tree diagrams. PCR-DGGE, polymerase chain reaction-denaturing gradient gel electrophoresis; A, negative control group; B, infection group; C, treatment group.

To analyze the similarity of the profiles in the different groups, tree diagrams were plotted and used for the comparison of the lower esophageal microbiota composition among the groups. According to the similarity coefficient, groups with samples showing a high similarity were classified into one class so that classes I, II and III were obtained. In class I, A2 mice exhibited relatively high similarity to A3 mice (0.46), but relatively low similarity to B3 mice (0.40). In class II, the groups with the highest similarity were C3 and C4, with a similarity coefficient of 0.88. Groups C5 and C6/C7 had a similarity coefficient of 0.82, and the similarity coefficient for groups C1 and C2 was 0.63. In class III, the highest similarity occurred between B1 and B2, with a coefficient of 0.62, followed by B5 and B9, with a similarity coefficient of 0.58. The lowest similarity coefficient was 0.56 for B6 and B7 (Fig. 6). These results showed that, with the exception of B3, bacteria in different mice could be separated on the evolutionary tree, suggesting that stable microbial community compositions existed in the lower esophagus, but with different compositions among the groups.

### Variations in the dominant flora species of the control, infection and treatment groups

**Figure 7:**
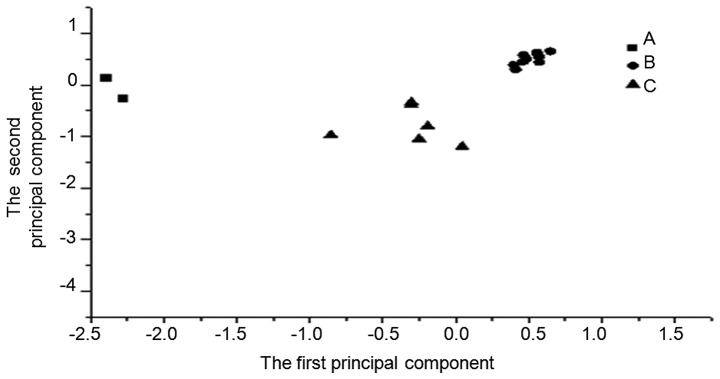
Denaturing gradient gel electrophoresis profiles of the lower esophagus in mice, as determined by principal component analysis. Principal component analysis diagrams for each group were drawn according to the values of the first and second principal component. The *x*-axis represents the first principal component (58.7%), The *y*-axis represents the second principal component (22.3%) and the percentage represents the main components. A, negative control group; B, infection group; C, treatment group.

To analyze the DGGE patterns in the different groups of mice, principal component analysis was performed. The data showed that the bacteria in the different groups gathered in different locations. Although the composition of flora species in the lower espohagus varied, the dominant species, and their relative contents, were similar in the control, infection and treatment groups (Fig. 7).

### Certain types of bacteria are found in the lower esophagus of all three groups, but certain bacteria are specific solely to the infection or control group

To analyze the bacterial species in the three groups, the mean OD30250 value was used as the cut-off value. Two unique bands (a1 and a2) were observed in group A and six (b1–b6) in group B; however, no unique bands were observed in group C. The three experimental groups shared three common bands (Fig. 6 and Table IV). The unique bands and common bands were amplified and BLAST analysis was performed to compare the sequences to determine the species of bacteria. These results suggested that certain types of bacteria were found in the lower esophagus of all three groups, but certain bacteria were specific solely to the infection or control group.

## Discussion

It has been reported that >200 types of bacteria can colonize in the lower esophagus (17). In the present study, DGGE fingerprint spectrum abundance confirmed that the lower esophagi of the studied mice each contained a microbiota composed of a large number of bacteria. The dominant types of bacteria included *Lactobacillus*, *Bacteroides*, *Staphylococcus*, *Escherichia coli*, *Klebsiella* and *Pseudomonas aeruginosa*.

In the present study, *H. pylori*-infected mouse models were constructed to analyze the DGGE profiles of the prokaryotic 16S rDNA V6 region in the lower esophagus of the infected and uninfected mice. It was found that both the number and species of the dominant bacteria in the lower esophagus increased significantly subsequent to the *H. pylori* infection. In addition, more complex types of bacteria, *Acinetobacter, Klebsiella, Enterobacter,* and various unknown species appeared. Previous studies have reported that *H. pylori* infection can change the microenvironment of the stomach, and inhibit or promote the growth of certain types of bacteria (18,19). Since the lower esophagus is closely connected to the stomach, changes in the microenvironment of the stomach may affect the microenvironment of the lower esophagus. Inflammation and mucosal injury of the lower esophagus in the infection and treatment groups indicated that *H. pylori* infection and antibiotic treatment could alter the microenvironment of the lower esophagus, causing changes in the composition of the lower esophageal microbiota.

Regarding the eradication of *H. pylori* infection using antibiotics, DGGE profile analysis of the 16S rDNA V6 region showed that the number of species in the lower esophageal microbiota was significantly reduced in the treatment group compared with the infection group, and no specific bacterial colonization was observed. This could be due to the antibiotics killing other bacteria as well as *H. pylori* or due to the antibiotic treatment changing the microenvironment in the lower esophagus to such an extent that the bacteria could not colonize.

Previous studies have suggested that lower esophageal sphincter pressure and lower esophageal acid reflux are the most important factors for changes in the pathology of the lower esophagus (20–23); however, decreased lower esophageal sphincter pressure may be due to the microbes in the lower esophagus. A study in mice showed that certain microbes in the lower esophagus are present from birth, while other microbial populations appear subsequent to colonization (20). Gram-negative bacteria have been reported to be the dominant bacteria causing acid reflux in the stomach; these bacteria cause relaxation of the lower esophageal sphincter predominantly through the induction of nitric oxide and enzymes (24).

In this study, following the eradication of *H. pylori* infection by antibiotics, the number of Gram-negative bacteria was not increased, but colonization with *Staphylococcus, Acinetobacter* and non-spore *Bacillus* was observed. The association between the changes in the lower esophageal microbiota and esophageal diseases therefore warrants further investigation.

## Figures and Tables

**Table I:** *Helicobacter pylori* colonization in different groups of mice.

Group	No. of mice	Positive result in urease test, n (%)	Positive result in culture, n (%)	Positive result in PCR, n (%)	Colonization n (%)	Eradication n (%)
A	15	0 (0)	0 (0)	0 (0)	0 (0)	-
B	15	14 (93)	15 (100)	14 (93)	15 (100)	-
C	15	1 (7)	1 (7)	1 (7)	-	14 (93)

Group A, negative control group; group B, infection group; group C, treatment group; PCR, polymerase chain reaction.

**Table II:** Degree of gastric mucosal lesions in mice.

Group	No. of mice	Mice without pathological changes, n (%)	Mice with inflammation, n (%)	Mice with mild ulcers, n (%)	Mice with large ulcers, n (%)
Infection	15	1 (7)	1 (7)	4 (27)	9 (60)
Treatment	15	1 (7)	3 (20)	8 (53)	3 (20)

**Table III:** Analysis of V6-denaturing gradient gel electrophoresis fingerprinting diversity.

Group	Diversity index (mean ± SD)	Margalef index (mean ± SD)	Pielou index (mean ± SD)
A	8.7±0.6	2.8±0.2	1.3±0.1
B	16.4±1.8	5.6±0.5	2.0±0.2
C	4.9±0.9	1.8±0.2	1.1±0.2

n=3 for each index. Group A, negative control group; group B, infection group; group C, treatment group.

**Table IV:** Analysis of bacteria species.

Band properties	Band no.	Species of bacteria	Similarity (%)
Specific for Group A	a1	*Lactobacillus*	98
	a2	*Bacteroides*	97
Specific for Group B	b1	*Staphylococcus*	99
	b2	*Acinetobacter*	97
	b3	*Bacteridium*	99
	b4	Uncultured bacterium	99
	b5	Uncultured bacterium	99
	b6	Uncultured bacterium	99
Common for Groups A, B and C	1	*Enterobacter*	99
	1	*Klebsiella*	99
	1	*Pseudomonas aeruginosa*	99

Group A, negative control group; group B, infection group; group C, treatment group.
